# Hybrid Heme Peroxidases from Rice Blast Fungus *Magnaporthe oryzae* Involved in Defence against Oxidative Stress

**DOI:** 10.3390/antiox9080655

**Published:** 2020-07-23

**Authors:** Marcel Zámocký, Anna Kamlárová, Daniel Maresch, Katarína Chovanová, Jana Harichová, Paul G. Furtmüller

**Affiliations:** 1 Department of Chemistry, Institute of Biochemistry, BOKU, University of Natural Resources and Life Sciences, Muthgasse 18, A-1190 Vienna, Austria; daniel.maresch@boku.ac.at (D.M.); paul.furtmueller@boku.ac.at (P.G.F.); 2 Laboratory of Phylogenomic Ecology, Institute of Molecular Biology, Slovak Academy of Sciences, Dúbravská cesta 21, SK-84551 Bratislava, Slovakia; anna.kamlarova@savba.sk (A.K.); katarina.chovanova@savba.sk (K.C.); jana.harichova@savba.sk (J.H.); 3 Institute of Experimental Medicine, Faculty of Medicine, Pavol Jozef Šafárik University, Trieda SNP 1, SK-04011 Košice, Slovakia

**Keywords:** oxidative stress, peroxidase-catalase superfamily, *Magnaporthe oryzae*, hybrid B heme peroxidase, carbohydrate binding domain

## Abstract

Hybrid B heme peroxidases are recently discovered unique oxidoreductases present solely in the fungal kingdom. We have investigated two typical representatives from *Magnaporthe oryzae*—one of the most dangerous phytopathogens known as a causal agent of the rice blast disease. First, we focused on native expression of two detected *hyBpox* paralogs by the means of reverse-transcription quantitative real-time PCR. Our results indicate a 7-fold induction of the *MohyBpox1* transcript in a medium with H_2_O_2_ and a 3-fold induction in a medium with peroxyacetic acid. For the *MohyBpox2* paralog the induction patterns were up to 12-fold and 6.7-fold, respectively. We have successfully expressed the shorter gene, *MohyBpox1*, heterologously in *Pichia pastoris* for detailed characterization. Observed biochemical and biophysical properties of the highly purified protein reveal that a typical HyBPOX is significantly different from previously investigated APx-CcP hybrids. This newly discovered secretory peroxidase reveals a Soret maximum at 407 nm, Q bands at 532 and 568 nm, CT band at 625 nm and a purity number of 1.48. Electron paramagnetic resonance (EPR) analysis suggests a mixture of high and low spin species in the ferric state dependent on calcium contents. Steady-state kinetic data reveal the highest peroxidase activity with ABTS, 5-aminosalycilate and efficient oxidation of tyrosine. MoHyBPOX1 as a fusion protein consists of two domains. The longer conserved N-terminal peroxidase domain is connected with a shorter C-terminal domain containing a carbohydrate binding motif of type CBM21. We demonstrate the capacity of MoHyBPOX1 to bind soluble starch efficiently. Potential involvement of hybrid peroxidases in the pathogenicity of *M. oryzae* is discussed.

## 1. Introduction

Peroxidases (EC 1.11.1.1–1.11.1.21, including 1.11.2.2; abbreviated mostly as POX) are ubiquitous oxidoreductases involved in the cleavage of reactive oxygen species containing a peroxide bond. Their sequences (including numerous novel ones from ongoing sequencing projects) are systematically classified in RedoxiBase [1] (http://peroxibase.toulouse.inra.fr) . The main division based on the prosthetic group involved in the catalytic reaction is in heme and non-heme containing enzymes. After previous comprehensive phylogenomic analyses four heme peroxidase superfamilies were identified that arose independently during the evolution [2]). Hybrid B peroxidases (HyBPOX) are unique fusion enzymes with their conserved N-terminal heme peroxidase domain clearly belonging to the peroxidase-catalase superfamily. Their rather variable C-terminal domains contain various sugar binding motifs [3]. Together with rather distantly related hybrid A peroxidases they represent two distinct turning points in the complex evolution within this largest known superfamily of heme *b* containing peroxidases [4]. Such rarely detected evolutionary hybrids within a particular protein (super)family might possess the capacity for a rapid adaptation of their robust fold to new selection pressures [5]. Thus, detailed investigations of hybrid peroxidases are of essential importance for the understanding of the complex evolution–structure–function relationships within the entire peroxidase-catalase superfamily by providing insight into yet missing intermediate developmental links. Hybrid A peroxidases containing heme were already investigated in sufficient details in several previous works (e.g., [6,7,8] they probably originated in ancestral protists and reveal polyphyletic traits). These enzymes represent real intermediates between highly specialised plant ascorbate peroxidases (APX) and yeast cytochrome *c* peroxidases (C*c*P). In contrast, their hybrid B counterparts remain still at the level of only putative predicted proteins [3,4]. From a recent in-depth phylogenetic analysis [2,9] it is however obvious that corresponding *hyBpox* genes have monophyletic origin and are present in almost all fungal genera. They are widely spread mainly among various phytopathogenic fungi. It is therefore important to investigate these peculiar peroxidases not only to reveal their potential “hybrid” character but also to demonstrate their physiological importance in various necrotrophic, biotrophic and hemibiotrophic fungi. We have analysed the expression of two *hyBpox* gene paralogs in the rice blast fungus *Magnaporthe oryzae* and chosen the isozyme MoHyBPOX1 for heterologous expression and detailed investigation of this protein as a typical representative for a very abundant subfamily as obvious from genomic analyses within recently published fungal genomes (e.g., [9,10,11,12]). The Sordariomycete *Magnaporthe oryzae* was positioned at first rank among the ten most important fungal pathogens worldwide [13] and besides preferentially attacking the rice it was recently described even as a causative agent of wheat blast [14]. An important aspect of the phytopathogenicity is a resistance of attacking fungus against the oxidative burst [15] that is accomplished by accumulation of reactive oxygen species (ROS) mainly from the action of plant NADPH oxidases in the apoplastic space of herbal host producing the superoxide radical [16]. As fused hybrid B peroxidases are with their peroxidase domain considered among potential ascomycetous and basidiomycetous antioxidant enzymes efficiently removing harmful ROS and contain additionally a sugar binding domain, they might play a significant role within host–pathogen interactions. In this work we could show at typical examples that recently discovered genes for hybrid peroxidases discovered in numerous fungal genomes can lead to the formation of peculiar functional peroxidases with unique protein properties that we present for the first time in this contribution.

## 2. Materials and Methods

### 2.1. Bioinformatic and Phylogenetic Analysis of Hybrid Peroxidases

The presence of two *hyBpox* paralogs in sequenced genomes of three typical representatives from the ascomycetous family Magnaporthaceae was detected with FGENESH 2.6 program of the Softberry suite [17]. Here analysed genes from the rice blast fungus *Magnaporthe oryzae* are annotated in RedoxiBase with accessions #2621 and #5356 and direct links to corresponding GenBank and UniProt files. Additionally, in taxonomically closely related fungi *Magnaporthe grisea* and *Gaeumannomyces graminis* pairs of *hyBpox* paralogs were detected (RedoxiBase accessions #14311 and #14381 for *M. grisea* genes and #11364 and #13889 for *G. graminis* counterparts). Multiple sequence alignment of complete sequences coding for 204 currently known hybrid heme peroxidases and further 46 sequences of the peroxidase-catalase superfamily was performed with Muscle program [18] using optimized parameters: gap open −0.8, gap extend −0.05, hydrophobicity multiplier 0.9 and the output was presented with GeneDoc software. Putative signal sequences of investigated peroxidases were analysed using the server SignalP 5.0 [19] and confirmed with BaCelLo [20]. Phylogenetic relationships of hybrid peroxidases were resolved using MEGA X software [21]. The following optimized parameters were applied: WAG+G+I+F model, 6 discrete gamma categories, partial deletion with 40% cutoff, nearest neighbour-interchange and branch swap filter was set to strong. For statistical support 100 bootstraps were used. Alternatively, the phylogenetic relations of the same data set were resolved with Mr Bayes version 3.2 [22] by using the Whelan and Goldman (WAG) model, invariant gamma rates and a relative burn-in of 25%. The run was performed over 2,500,000 generations.

### 2.2. Cultivation of the Fungus and Detection of Native Expression of MohyBpox1 and MohyBpox2 Gene Paralogs

The fungus *Magnaporthe oryzae* CBS 131,616 was obtained from Centraalbureau voor Schimmelcultures (CBS, Utrecht, The Netherlands). This fungus was grown in MPG medium (20 g malt extract, 1 g peptone and 20 g glucose for 1 L) at 28 °C and 120 rpm in New Brunswick Innova 40/40R shaker for 3–6 days. Cultures with the addition of H_2_O_2_ (10 mM and 5 mM), peroxyacetic acid (5 mM) or paraquat (5 mM), Cd^2+^ ions (25 mM) and t-butyl hydroperoxide (10 mM) were grown under the same conditions as non-induced controls. The induction of oxidative stress was tested with all mentioned stressors stepwise in the interval of 10–60 min and afterwards the mycelia were collected by sterile filtration. For the detection of native expression of hybrid peroxidases total RNA of this fungus was isolated from all cultures in various growth phases with RNeasy Plus Mini Kit from Qiagen (Hilden, Germany) using the corresponding quick-start protocol on fresh or −80 °C frozen mycelia. Obtained RNA samples were directly subjected to real-time quantitative PCR method (RT-qPCR) in AriaMx6 device (Agilent Technologies, Santa Clara, USA) using the Brilliant III Ultra-Fast SYBR Green Master Mix (Agilent Technologies, Santa Clara, USA). Optimized conditions for RT-qPCR were: first step incubation for 10 min at 50 °C followed by 3 min at 95 °C and two step PCR for 5 s at 95 °C and 15 s at 60 °C repeated 40 times and a final step of 1 cycle for 30 s at 95 °C and 60 °C, respectively. Obtained output was analysed with Agilent AriaMx Software version 1.0 and the level of peroxidase gene expression was calculated from relative quantification with the formula 2^−ΔΔCt^ known as the method of Livak and Schmittgen [23] where Ct is the threshold cycle. The amount of peroxidase mRNA target was normalized to the constitutively expressed ITS-1 region of *M. oryzae*. Alternatively, for analysis of longer mRNA transcripts corresponding cDNA was produced with the first strand cDNA synthesis kit using the oligo-d(T)_23_ primer. RT-PCR was performed as follows: 2 min at 95 °C followed by 35 cycles for 30 s at 95 °C, 1 min at the corresponding annealing temperature (dependent on used primers listed in Supplementary Table S1) and 40 s at 68 °C using the AccuPrime Pfx DNA polymerase (Life Technologies, Carlsbad USA). Obtained RT-PCR products were purified and sequenced with the Sanger method at GATC Biotech, Konstanz, Germany.

### 2.3. Heterologous Expression of MoHyBPOX1 in Pichia pastoris BG11 (AOX1) Strain

Synthetic gene coding for MoHyBPOX1 with additional N-terminal strep-tag and C-terminal 6 His-tag (sequence in GenBank file MT656060) was produced with codon optimization for *Pichia pastoris* expression at ATUM (Menlo Park, CA, USA) and cloned in the pJ912 shuttle vector carrying zeocin resistance. For the purpose of *Pichia pastoris* electroporation it was linearized with the restriction endonuclease *SwaI* (New England Biolabs, Ipswich USA) and transformed into *Pichia pastoris* strain BG11 (AOX1, mutS Bisy, Austria) via electroporation on the BioRad electroporator by using the program Fungi—Sc2 in 2 mm cuvettes. Transformants were selected on YPD plates (10 g/L yeast extract, 20 g/L peptone, 10 g/L glucose (dextrose) and 15 g/L agar) supplemented with zeocin (up to 100 mg/L). In selected transformants the integration of the linearised plasmid into genomic DNA was controlled with genomic PCR using *hyBPOX1*-specific primers listed in Table S1.

For the expression of the synthetic *MohyBpox1* gene overnight cultures of PCR-verified *Pichia pastoris* clones were first cultivated in YP medium supplemented with 1% glycerol at 28 °C and 180 rpm. Ten millilitre aliquots of these precultures were further inoculated in 200 mL of fresh YP-glycerol medium supplemented with 0.4 mL of 0.02% biotin stock solution in baffled shaken flasks and incubated at 28 °C, 180 rpm. After complete consumption of glycerol (24 h) methanol was added to 0.5% final concentration for the induction of recombinant peroxidase production under the control of AOX1 promoter (in pJ912 vector). The growth was continued for a further 48 h at 25 °C and 180 rpm with daily addition of methanol (up to 1% final concentration) thus forming the YPM growth medium. Hemin solution (Sigma Aldrich stabilized at pH 9.0) was added to a final concentration of 10 µM shortly after the second addition of methanol.

### 2.4. Purification of Recombinantly Produced MoHyBPOX1

Cultures of selected clones in *Pichia pastoris* BG11 after their induction with 1% methanol in YPM and 72 h growth were separated from the cultivation medium by centrifugation for 10 min. at 3000× *g*. The obtained supernatant was used for the precipitation of proteins with ammonium sulfate in two steps. The first step to 31% saturation (170 g of ammonium sulfate added stepwise at 0 °C to 1 L of protein solution within 30 min) separated after 20 min of centrifugation at 45,000× *g* mainly various *Pichia* proteins and cell debris in the formed pellet leaving the recombinant protein in the supernatant. The second step was performed up to 82% saturation (343 g of ammonium sulfate added stepwise at 0 °C to 1 L of remained protein solution within 30 min.) leading to the precipitation of almost all remaining proteins. Recombinant hybrid peroxidase was collected in the pellet also by 20 min centrifugation at 45,000× *g*.

This second pellet was then resuspended in 50 mL MCAC-A buffer (50 mM sodium phosphate, 0.5 M NaCl, pH 8.0 with freshly added 1 tablet of protease inhibitor cocktail—Sigma S8830) and after a brief centrifugation at 45,000× *g* the obtained clear supernatant was immediately loaded on 30 mL Ni-NTA agarose column (Qiagen, Germany) equilibrated in the same buffer (without protease inhibitors). After washing this column with at least 5 column volumes of MCAC-A buffer His-tagged proteins were eluted with a linear gradient of 0–500 mM imidazole in 50 mM sodium phosphate and 500 mM NaCl, pH 8.0 (MCAC-B buffer). Obtained 3 mL fractions were analysed by UV-vis spectroscopy and fractions with a well resolved Soret peak were pooled together and concentrated in Vivaspin 30 kDa devices (GE Healthcare, Little Chalfont, Great Britain) for 20 min at 4000× *g*. This was followed by PD10 column desalting (GE Healthcare) in 20 mM Tris-HCl buffer, pH 7.5 containing 0.5 mM Ca^2+^ for stabilization of the recombinant peroxidase. For final purification a 25 mL DEAE-Sepharose column (GE-Healthcare) equilibrated with solution A (20 mM Tris-HCl and 0.5 mM CaCl_2_, pH 7.5) was used. The strongly bound peroxidase sample was washed with at least 5 column volumes of solution A, afterwards a linear gradient to 100% solution B (2 M NaCl in 20 mM Tris-HCl and 0.5 mM CaCl_2_, pH 7.5) was applied. Final fractions containing the peroxidase (detected in UV-vis spectrum) were collected together, desalted with the PD10 column and again concentrated with Vivaspin 30 kDa device (GE Healthcare) under the same conditions as described above.

Protein concentration was determined with the Bradford method at 595 nm with a 5-fold diluted Roti-Quant Bradford solution (Roth, Germany) from a calibration curve constructed with bovine serum albumin (with a concentration range of 0.1–1.0 mg/mL) as a standard. The control of purified hybrid peroxidase samples was performed with SDS-PAGE run as 4–12% gradient gels (Life Technologies) that were either stained with Coomassie Brilliant Blue or used for Western blotting. The detection of transferred proteins on the nitrocellulose membrane (Amersham) was performed using a monoclonal mouse Penta-His Antibody (Qiagen 34660) diluted in Tris-buffered saline (TBS) buffer pH 7.4 to a final concentration of 50 ng/µL and a secondary antibody against mouse IgG conjugated with alkaline phosphatase (Sigma A-3562) diluted from the original stock solution in TBS buffer, pH 7.4, to a final concentration of 5 ng/µL. The colorimetric detection of positive bands was performed in a solution containing 5-bromo-4-chloro-3indolyl phosphate (BCIP) and nitro blue tetrazolium (NBT, both from Promega) for 10 min according to the manufacturer’s instructions.

### 2.5. Deglycosylation of Purified Hybrid Peroxidase

Deglycosylation was performed with Endo Hf and PNGase F (New England Biolabs) following the recommended protocol of the producer. Of purified MoHyBPOX1 20 µg were mixed with the corresponding glycoprotein denaturing buffer in a final volume of 10 µL. This protein was denatured for 5 min at 96 °C and the reaction volume was afterwards increased to 20 µL by adding 10× G7 reaction buffer, 10% NP40 and sterile distilled H_2_O. This mixture was incubated with 1 µL PNGase F for 1 h at 37 °C. Alternatively, denatured MoHyBPOX1 sample was increased to 20 µL by adding 10× G5 reaction buffer, sterile distilled H_2_O and 1 µL of the EndoH enzyme. Also, this mixture was incubated for 1 h at 37 °C. Afterwards obtained samples were analysed with SDS-PAGE under conditions described above.

### 2.6. Protein Identification and Peptide Analysis Using LC-ESI-MS

The relevant protein bands were cut out and digested in gel. S-alkylation with iodoacetamide and digestion with sequencing grade modified trypsin (Promega) were performed overnight. The peptide mixture was analysed using a Dionex Ultimate 3000 system directly linked to a QTOF instrument (maXis 4G ETD, Bruker, Hanau Germany) equipped with the standard electrospray ionisation (ESI) source (CaptiveSpray nanoBooster also from Bruker, Hanau Germany, respectively) in the positive ion, data dependent acquisition (DDA) mode (=switching to the MSMS mode for eluting peaks). MS scans were recorded (range: 150–2200 *m/z*) and the 6 highest peaks were selected for fragmentation. Instrument calibration was performed using the ESI calibration mixture (Agilent). For separation of the peptides a Thermo BioBasic C18 separation column (5 µm particle size, 150 mm × 0.320 mm) was used. A gradient from 95% solvent A and 5% solvent B (solvent A: 65 mM ammonium formiate buffer, B: 100% acetonitrile) to 32% B in 45 min was applied, followed by a 15 min gradient from 32% B to 75% B, at a flow rate of 6 µL/min. For the measurements in nano-mode a Thermo Acclaim PepMap300 RSLC C18 separation column (2 µm particle size, 150 mm × 0.075 mm) was used with a Thermo Acclaim PepMap µ-precolumn. A gradient from 5% solvent B (solvent A: 0.1% formic acid in HQ-water, solvent B: 0.1% formic acid in acetonitrile) to 32% B in 60 min was applied, followed by a 10 min gradient from 32% B to 70% B that facilitates elution of large peptides, at a flow rate of 0.3 µL/min.

The analysis files were converted using Data Analysis 4.0 (Bruker) to XML files, which are suitable to perform MS/MS ion searches with MASCOT (embedded in ProteinScape 3.0, Bruker) for protein identification. Only proteins identified with at least 2 peptides with a protein score higher than 80 were accepted. For the searches the reviewed UniProt database was used. Peptide MS/MS data were evaluated against the target sequence using X! Tandem (http://www.thegpm.org/tandem/) with settings allowing residue modifications: oxidation of M, W and deamidation of N, Q. Further, isotope error was considered; fragment type was set to monoisotopic; refinement was used with standard parameters and fragment mass error of 0.1 Da and ± 7 ppm parent mass error was applied.

### 2.7. N-Glycan Release and LC-ESI-MS Analysis of Free N-Glycans

An aliquot of the peptide mixture (2.6) was treated with PNGaseA (ProZyme, Santa Clara, USA) overnight and further reduced with sodium borohydride for at least 4 h at room temperature. The samples were purified with HyperSep™ Hypercarb™ SPE Cartridges (Thermo Scientific, Waltham, USA). Mass spectrometric analysis was performed using an amaZon speed ETD ion trap (Bruker), which was operated in the data dependent acquisition mode (ES+, target mass 800 *m/z*, ICC 100000, 200 ms, enhanced resolution). Auto MS2 was performed on the 4 dominant precursor peaks (Auto MS2 threshold abs 40000) by collision-induced dissociation (CID). For separation of the released N-glycans a Dionex Ultimate 3000 system with a Hypercarb PGC column (100 mm × 0.320 mm, 5 µm, Thermo Scientific) was used directly coupled to the mass spectrometer. A gradient from 99% solvent A and 1% solvent B (solvent A: 65 mM ammonium formiate buffer, B: 100% acetonitrile) to 62.5% B in 30 min was applied at a flow rate of 6 µL/min.

### 2.8. UV-Vis and Electronic Circular Dichroism Spectroscopy

UV-vis spectrum of affinity-purified recombinant MoHyBPOX1 was recorded on Hitachi U-3900 spectrophotometer at 25 °C with a scan speed of 60 nm/min in the interval between pH 5.0 to pH 8.0. The molar absorption coefficient at 280 nm determined at Expasy Protparam server from the complete sequence of recombinant MoHyBPOX1 protein with a hexa His-tag was determined as ε_280 nm_ = 39,795 M^−1^ cm^−1^ (all Cys in cystines). The molar absorption coefficient at Soret maximum was determined as ε_407 nm_ = 73,100 M^−1^ cm^−1^ in this work on series of dilutions for the best affinity and ion-exchange purified fractions. As a quality criterion for each heme peroxidase sample, the Reinheitszahl (RZ), i.e., the ratio between A_Soret_max_/A_280 nm_ was recorded—the theoretical maximal RZ value of MoHyBPOX1 from both molar absorption coefficients shall be RZ = 1.84.

Electronic circular dichroism (ECD) spectra were recorded on Chirascan spectrophotometer (Applied Photophysics, Leatherhead, U.K.) flushed with nitrogen with a flow rate of 5 L/min. Following parameters were used for recording far-UV spectra: range 260–190 nm, path length of 1 mm, spectral bandwidth 3 nm, step size 1 nm and scan time 12 s per point. Thermal unfolding was monitored by stepwise increasing the temperature for 1 °C/min in the range 20–90 °C with the spectral bandwidth 0.5 nm and scan time 12 s per point. The melting temperature (*T*_m_) was determined from sigmoidal fitting of obtained profile using Pro-Data Viewer software from Applied Photophysics (Version 4.1.9).

### 2.9. Electron Paramagnetic Resonance

Electron paramagnetic resonance (EPR) study was performed on a Bruker EMX continuous wave spectrophotometer. This device operating at X-band (9 GHz) was equipped with a high sensitivity resonator and an Oxford Instruments ESR900 helium cryostat operating at 10 K. Spectra were recorded under non-saturating conditions using 2 mW microwave power, 100 kHz modulation frequency, 1 mT modulation amplitude, 41 ms conversion time, 41 ms time constant and 2048 points. For the measurement, a 100 µL sample of 50 µM affinity purified and desalted MoHyBPOX1 was filled in the Wilmad quartz tube that was flash frozen in liquid nitrogen. In order to remove O_2_ the tube was flushed with argon while the sample was kept frozen on dry ice.

### 2.10. Thermodynamic and Kinetics of Cyanide Binding

The binding of sodium cyanide on purified hybrid peroxidase was studied by stepwise addition of cyanide stock solution (10 mM) to 5 µM purified MoHyBPOX1 peroxidase and recording the shift in the electronic spectrum after each mixing. The range between 10 µM and 2 mM cyanide (final concentration) was investigated. The dissociation constant was calculated from the Hanes plot.

### 2.11. Determination of the Peroxidatic Activity

Peroxidatic activity was determined spectrophotometrically by using either hydrogen peroxide or peroxyacetic acid as electron acceptors (both 1 mM) and ABTS (2,2′-azino-bis-3-ethylbenzothiazoline-6-sulfonic acid, ε_414 nm_ 31.1 mM^−1^ cm^−1^); guaiacol (ε_470 nm_ 26.6 mM^−1^ cm^−1^), L-Dihydorhyphenylalanine (DOPA, ε_470 nm_ 3.6 mM^−1^ cm^−1^), pyrogallol (ε_470 nm_ 11.3 mM^−1^ cm^−1^), 3,3′,5,5′ tetramethylbenzidine (TMB, ε_652 nm_ 2.8 mM^−1^ cm^−1^), ascorbate (ε_290 nm_ 2.8 mM^−1^ cm^−1^) or Mn^2+^ cations (ε_238 nm_ 6.5 mM^−1^ cm^−1^) as one-electron donors. Peroxidatic activity was also recorded fluorometrically by using tyrosine as a one-electron donor. In this case the excitation wavelength was 325 nm and the emission wavelength 405 nm. All kinetic profiles were recorded either on a Hitachi U-3900 spectrophotometer or Hitachi F-4500 spectrofluorimeter at constant temperature (30° C) with permanent stirring during all measurements.

### 2.12. Detection of Binding of Soluble Starch on Purified MoHyBPOX1 Protein

A specific binding of starch to the recombinant highly purified MoHyBPOX1 protein was investigated. First, 0.25 mL of a 2.0% soluble starch solution (Merck CAS-Nr. 9005-84-9) with the formula (C_12_H_22_O_11_)_n_ was mixed with 1.0 mL of 7 µM MoHyBPOX1 in 20 mM Tris HCl 0.5 mM CaCl_2_ buffer pH 7.5 followed by intensive shaking at room temperature for 60 min. Afterwards, the unbound starch was separated from MoHyBPOX1 protein over a 30 mL Superdex_200_ column (GE Healthcare) in the same buffer and only fractions containing the protein (detected with the Bradford method) were collected. In these fractions the concentration of starch was determined colorimetrically with the method of Jacobsen [24] after forming a KI/I_2_-starch complex from a calibration curve recorded at 610 nm from a set of standards with stepwise increased concentration of starch.

## 3. Results and Discussion

### 3.1. Phylogenomic Analysis and Quantification of Native Expression for MohyBpox1 and MohyBpox2 Gene Paralogs with RT-qPCR Methodology

**Figure 1:**
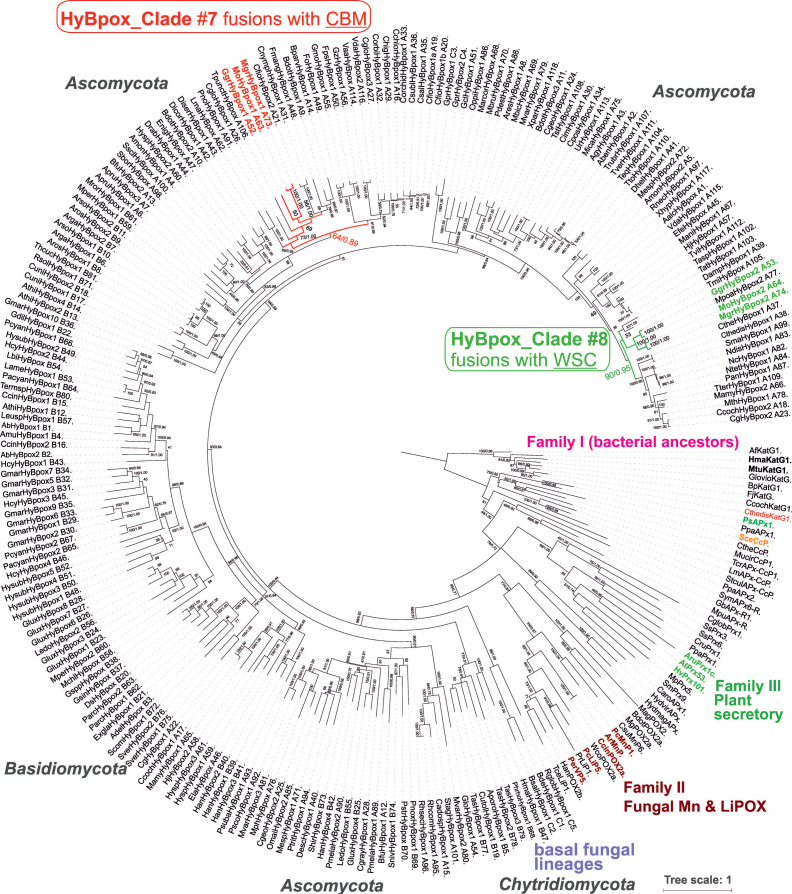
Phylogenetic position of sequences coding for peroxidases from phytopathogenic representatives of the fungal family *Magnaporthaceae* analysed in an unrooted phylogenetic tree for all known hybrid B peroxidase sequences and selected representatives from all other known clades of the peroxidase-catalase superfamily. Presented is a tree topology obtained with the maximum likelihood method of the MEGA X package [21]. Whelan and Goldman (WAG) as the best substitution model for this dataset of 250 complete protein sequences was used with 5 gamma categories and invariant sites and 100 bootstrap replicates. A very similar tree topology was obtained also with the Mr Bayes approach [22] with a relative burn-in of 25% performed over 1,500,000 generations. Numbers in the nodes represent bootstrap values and posterior probabilities, respectively (presented are only values above 33/0.5). All here used abbreviations of peroxidases correspond with RedoxiBase [1] (http://peroxibase.toulouse.inra.fr/).

**Figure 2:**
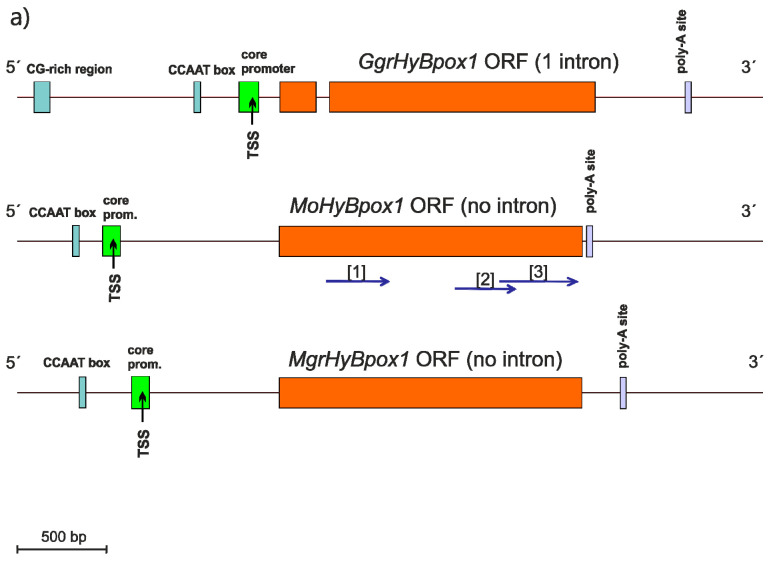

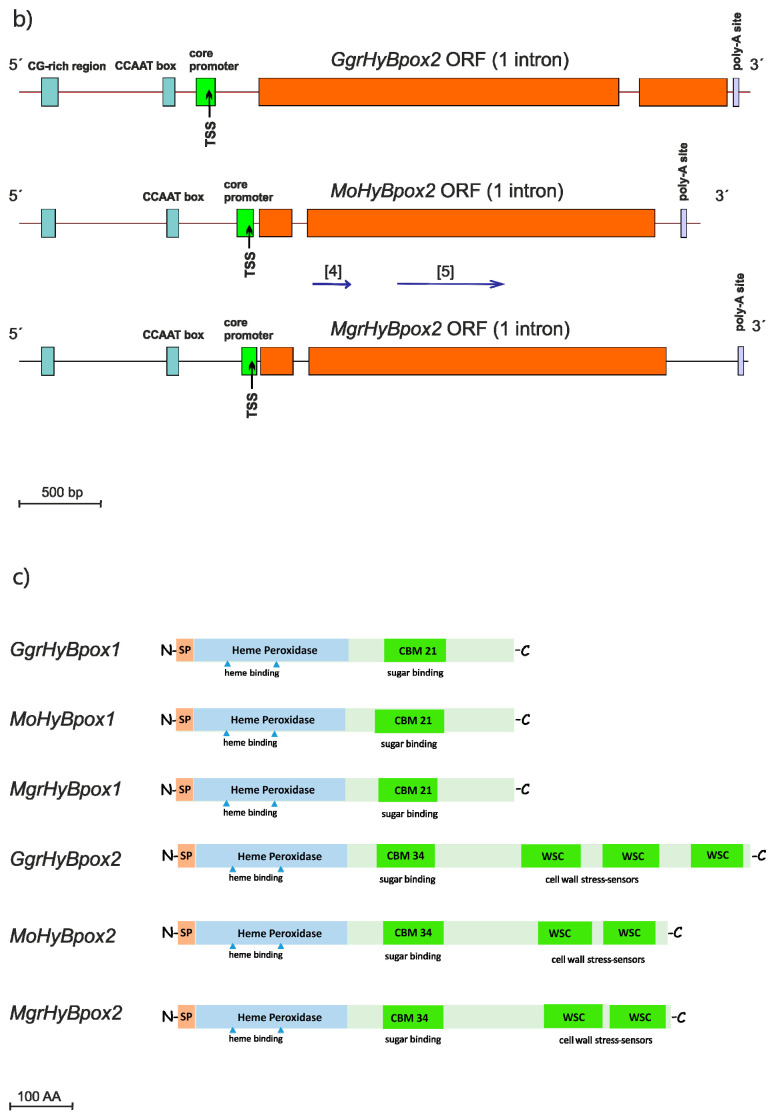
Genomic regions coding for closely related Hybrid B heme peroxidases from phytopathogens of the Magnaporthaceae family. Schematically presented are corresponding paralog genomic sequences for (**a**) *GgrhyBpox1* from *Gauemannomyces graminis* (strain R3-111a-1)*,* for *MohyBpox1* from *Magnaporthe oryzae* (strain 70-15) and for *MgrhyBpox1* from *Magnaporthe grisea* (strain DS0505); (**b**) for *GgrhyBpox2*, *MohyBpox2* and *MgrHyBpox2*, respectively. Experimentally obtained mRNA fragments coding for regions of *MohyBpox1* are highlighted with blue arrows. Numbers in brackets correspond to GenBank EST accessions [1] JZ969880, [2] JZ970417 and JZ970418 and JZ970399, [3] JZ969979, [4] JZ97400 and JZ970401 and JZ970402 and [5] JZ970248 and JZ970250. Prediction of promoter elements, introns and poly-A sites was performed with FGENESH 2.6 suite [17]. (**c**) Translation products of corresponding mRNAs with schematic presentation of conserved peroxidase and variable non-peroxidase domain motifs. Drawn to scale according to predictions.

A systematic genomic analysis reveals that newly discovered genes coding for Hybrid B peroxidases (abbreviated as *hyBPOX* to differ them from other peroxidase genes) are present in almost all already sequenced fungal genomes including also the basal fungal lineages, e.g., [9,10,11,12]. A previous phylogenetic analysis revealed that this monophyletic gene family possessing its direct ancestor in the large peroxidase-catalase superfamily [2] could be further divided into seven subfamilies [4]. We have chosen two isozymes of hybrid B peroxidase present in the phytopathogenic rice blast fungus *Magnaporthe oryzae* and their counterparts from closely related pathogenic fungi *Mangaporthe grisea* and *Gaeumannomyces graminis* for a detailed investigation because according to their domain composition they are typical representatives of two distinct types of these enzymes in plant pathogenic fungi with a possible impact on plant–fungus interactions. Figure 1 reveals their phylogenetic position in an updated phylogenetic tree comprising of 250 complete sequences chosen from the whole peroxidase-catalase superfamily. Whereas *Magnaporthaceae* HyBPOX1-representatives were located within clade #7 (labelled red in Figure 1), HyBPOX2-paralogs were located in clade #8 (labelled green) with a high bootstrap support. There appeared a quite long evolutionary distance between these two peroxidase paralogs. Thus, they diverged rather early from each other in the ancestral fungal genome probably even before the segregation between Ascomycota and Basidiomycota. We analysed the corresponding genomic regions (Figure 2a,b). Core promoter regions including the transcription start sites (TSS), typical CCAAT boxes and 3’ motifs for polyadenylation sites for all here presented *hyBpox* genes were detected with FGENESH [17] and revealed small variations between closely related species. Four of these genes contained one short intron and the remaining two are intronless. All six translated (and spliced) open reading frames of these genomic regions code for multidomain proteins. Obviously, *Magnaporthaceae* HyBPOX1s contain N-terminal heme peroxidase domain classified in Pfam00141 and C-terminal domain containing a sugar-binding motif classified as Carbohydrate Binding Module CBM21. In contrast, HyBPOX2s from the same genomes contain besides the N-terminal peroxidase domain (of the same length and also classified in Pfam00141) a single middle domain with a closely related CBM34 sugar-binding motif. Further, they possess also two or even three C-terminal water soluble carbohydrate binding (WSC) domains (Figure 2c) known as cell-wall stress sensors [25].

**Figure 3:**
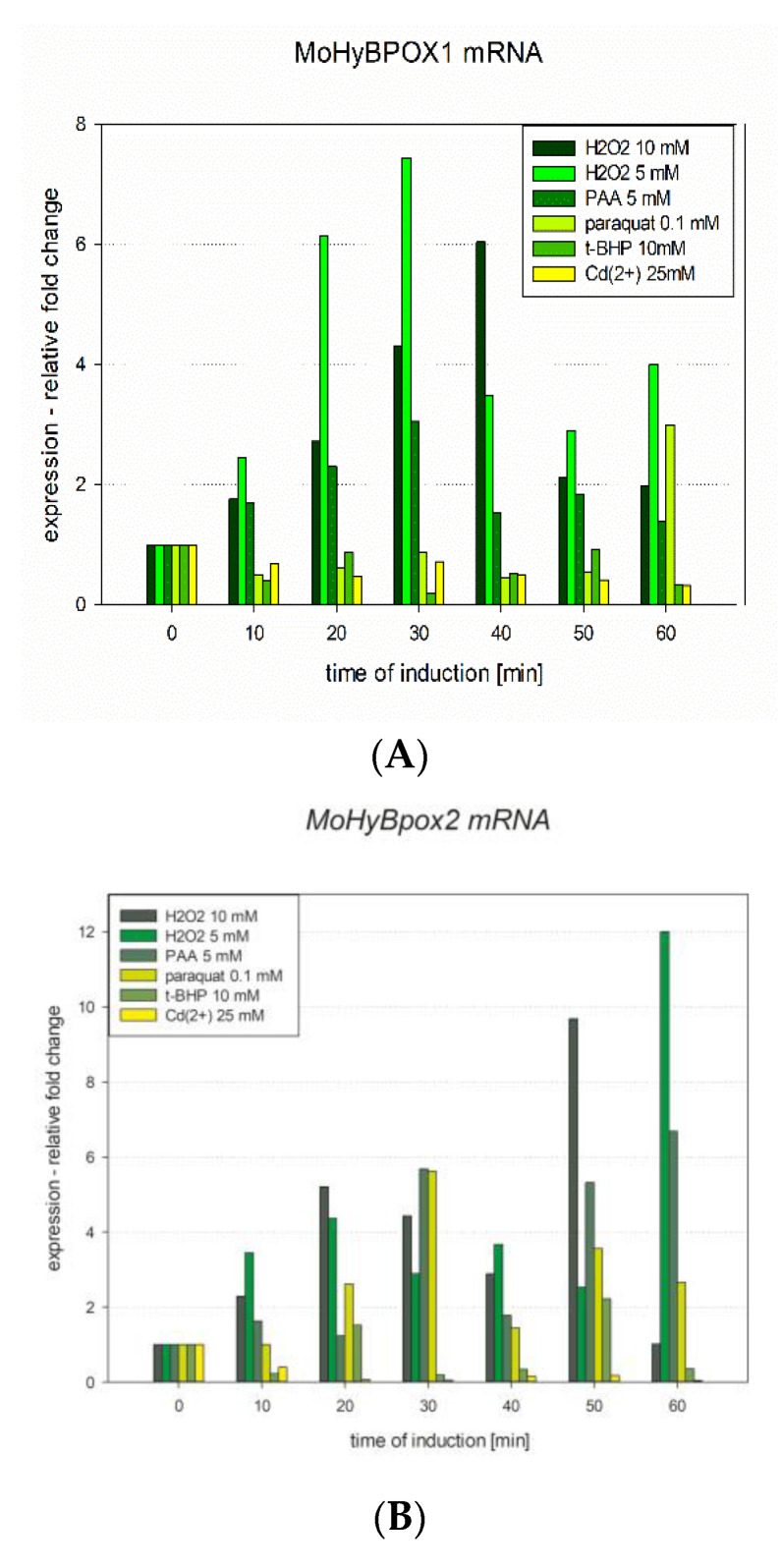
Inducible expression of two gene paralogs of hybrid peroxidase gene in *Magnaporthe oryzae* detected and quantified with RT-qPCR methodology: (**A**) time traces for changes in the production of *MohyBpox1-mRNA* with 5 different oxidative stress inducers using Mor4-specific transcript (Supplementary Table S1) and (**B**) time traces for changes in the production of *MohyBpox2*-mRNA with 5 different oxidative stress inducers using Mor2-specific transcript (Supplementary Table S1).

Results of the genomic analysis prompted us to verify the native expression of these yet unknown genes. First, we prepared mRNA libraries from MPG-grown *Magnaporthe oryzae* cultures induced either with 10 mM H_2_O_2_ or with 5 mM H_2_O_2_. Further similar mRNA libraries were prepared with an induction caused by the addition of 5 mM peroxyacetic acid, 0.1 mM paraquat, 25 mM Cd^2+^ ions and 10 mM tert.-butylhydroperoxide to the same fungus. For all these samples also a control mRNA library was prepared with no induction in the same growth medium. Then, we prepared the corresponding cDNAs and confirmed predicted coding regions in all parts of the *MohyBpox* genes after sequencing of obtained RT-PCR products (Table 1 and Figure 2a,b where obtained mRNA fragments are positioned). To quantify the level of expression of these genes under various oxidative stress conditions we have applied RT-qPCR methodology on all prepared *mRNA* libraries. Relative quantifications obtained with the formula 2^−ΔΔCt^ [23] were normalized by using the constitutively expressed ITS-1 gene for both investigated *hyBpox* paralogs. Resulting values for all investigated mRNA samples are presented in Figure 3. The highest level of induction was observed with 5 mM H_2_O_2_ induced-samples for both gene paralogs. Time traces show that the maximum of *MohyBpox1* expression, namely 7-fold was achieved for the sample collected 30 min after hydrogen peroxide addition (Figure 3A). Similar profile was achieved also with 10 mM hydrogen peroxide but the 6-fold maximum for *MohyBpox1* was shifted to the 40 min induced sample. Additionally, peroxyacetic acid, added to 5 mM final concentration in the growth medium caused a very similar but lower level of induction with 3-fold maximum in the 30 min sample but all other oxidative stressors did not cause any induction of *MohyBpox1* transcription indicating a possible different pathway of regulation for them. *MohyBpox2* exhibited a more complex profile (Figure 3B). Time trace was highest again with the induction caused by 5 mM H_2_O_2_ but there was a local 4.5-fold increase at 20 min and the maximum of induction namely 12-fold increase was observed in the 60 min sample. For 10 mM H_2_O_2_ series corresponding 9.5-fold maximum was shifted to 50 min. For 5 mM peroxyacetic acid the achieved 6.7-fold maximum was also in the 50 min sample of induction and from all other oxidative stressors only 0.1 mM paraquat caused a 5.7–3.5-fold induction of transcription for this longer gene variant in time traces 30 min and 50 min, respectively. We have shown that fungal cells from all here induced samples were viable on MPG-agar plates after the treatment with inductors, so the eventual decrease in the expression in some samples cannot be attributed to the death of cells treated with various oxidative stress inducers.

### 3.2. Heterologous Expression of Recombinant MoHyBPOX1 in Pichia pastoris

As the fungi from the family Magnaporthaceae containing presented hybrid peroxidase genes are among the most dangerous phytopathogens known [13], we decided to produce a recombinant protein of MoHyBPOX1 as a typical example via secretion from methylotrophic yeast *Pichia pastoris*. The synthetic gene was codon-optimized for this expression system and allowed higher and regulated production of this novel peroxidase for a detailed investigation of its properties. Control of the integration of synthetic *MohyBpox1* gene after electroporation into chromosome of *Pichia pastoris* BG11 (mutS) was achieved with genomic PCR of zeocin transformants using *MohyBpox1* specific primers (Supplementary Table S1 and Supplementary Figure S1A). No known genes for hybrid peroxidases (or closely related genes) exist in the natural, non-transformed yeast genome so this positive result indicates the successful integration of our synthetic gene in the chromosome of *Pichia pastoris*. Four best clones from the screening of transformed *Pichia pastoris* BG11 (mutS) strain that were proven as positive, namely: MoHyBPOX13am, -13fm, -19gm and -110cm were selected for the process of secretory overexpression and His-tagged purification of recombinant peroxidase as described in Section 2.4.

The purification of secreted hybrid B peroxidase was achieved via fractionated ammonium sulfate precipitation followed by His-tag metal chelate affinity chromatography (MCAC) purification on the NiNTA column and finally the best pooled fractions were purified on an ion exchange chromatography on DEAE Sepharose. Mass spectrometry detection of the peptides originating from the recombinant protein MoHyBPOX1 secreted from *Pichia pastoris* cells was analysed on obtained protein bands cut from a 4–12% SDS-PAGE gel (Supplementary Figure S1B) in the range of 60–75 kDa (corresponds to the size of glycosylated monomer) and digested with trypsin. Sequence coverage for the entire protein sequence coding for this heme peroxidase (Supplementary Figure S2A) was calculated as 70.74% and recorded peptides were distributed along the whole coding sequence. This result indicates that the entire protein of this novel peroxidase was secreted from *Pichia* cells and is stable against degradation by proteolytic attack. The MS1 sum-spectrum of this two-domain protein shows a characteristic high mannose type glycosylation profile that is quite typical for the secretory expression of fungal peroxidases in the yeast *Pichia pastoris* (Supplementary Figure S2B). Basic properties of the newly discovered hybrid B heme peroxidase are listed in Supplementary Table S2.

### 3.3. Spectroscopic Properties of Hybrid B Peroxidase

**Figure 4:**
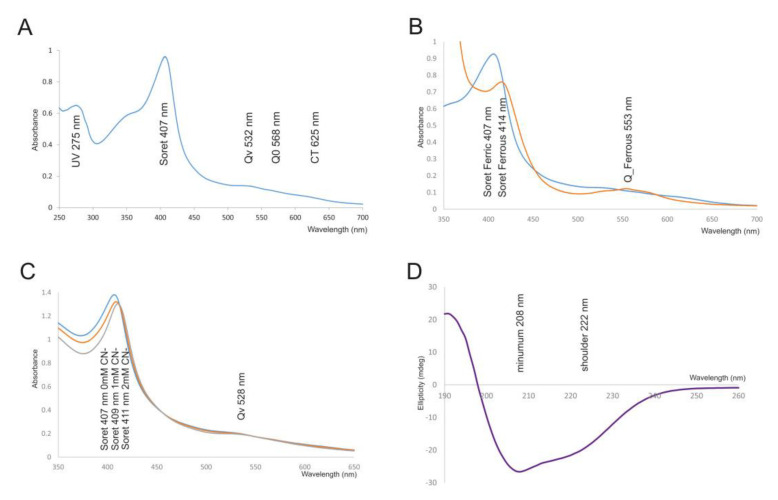
Spectroscopic features of highly purified MoHyBPOX1 heme protein. (**A**) UV-vis spectrum of the purified ferric form with Soret maximum at 407 nm and RZ value of 1.48 revealing a very high level of purity for this heme protein. (**B**) Reduction to ferrous form with the addition of sodium dithionite resulting in a shift of Soret maximum to 414 nm and occurrence of an additional visible band at 553 nm. (**C**) Stepwise titration of purified MoHyBPOX1 with increasing concentration of NaCN leading to a shift in Soret maximum from 407 to 411 nm. (**D**) Far-UV ECD spectrum of affinity purified MoHyBPOX1 protein recorded between 190 and 260 nm with a typical minimum at 208 nm and a shoulder at 222 nm. The content of the secondary structure elements is calculated in Table 2.

MCAC and ion exchange purified and desalted hybrid B peroxidase from *M. oryzae* revealed typical spectral features at pH 7.0 (Figure 4A): a Soret maximum at 407 nm, Q bands at 532 nm, 568 nm and a charge transfer band at 625 nm and showed only small variations of Soret maximum in the interval of pH from 5.0 to 8.0 (Supplementary Table S2). Typical RZ value for different purifications varied from 1.29 to 1.48. Soret maximum for this novel peroxidase was only slightly red-shifted if compared with well-known horseradish peroxidase (HRP) or soybean peroxidase (both at 403 nm as typical Family III representatives). However, it was nearly identical with Family II fungal peroxidases, e.g., *P. chrysosporium* manganese peroxidase (maximum at 406 nm) or lignin peroxidase (407 nm) or the versatile peroxidase from *P. eryngii* (406 nm, also a Family II peroxidase—detailed overview in [26]). Therefore, we can suppose that the spectroscopic signatures of hybrid B peroxidases were more similar to Family II of the peroxidase-catalase superfamily than to Family III. For Family I peroxidases the Soret maximum was mostly around 404 nm (e.g., [27]).

Changes in the UV-vis spectrum upon addition of a small amount of Na_2_S_2_O_4_ causing the reduction of iron in the heme from ferric to the ferrous state were recorded under the same conditions of temperature, pH and ionic strength and a significant shift from 407 to 414 nm was observed (Figure 4B). This shift of the Soret band was accompanied with the occurrence of an additional small band in the visible region at 553 nm. Such spectral changes are well comparable with similar spectral shifts of other members of the peroxidase-catalase superfamily (e.g., [27,28] and reveal the ability of rather easy reduction of ferric iron in the active centre of Hybrid B peroxidase).

Titration of MoHyBPOX1 with NaCN caused typical transition from a high-spin (s = 5/2) to low-spin (s = 1/2) complex with the characteristic red shift of the Soret band from 407 to 411 nm (Figure 4C). The calculated dissociation constant of this complex was *K*_D_ = 71 µM (Supplementary Table S2), which was significantly higher than dissociation constants for the closest related catalase-peroxidases from the same fungus (5–47-fold higher, e.g., [27,28]. It can be thus reasoned that the active centre of hybrid B peroxidase was not so easily accessible for cyanide ions as for intracellular heme peroxidases probably due to observed intensive glycosylation at the surface of this protein (cf. Section 3.2).

Far-UV ECD spectrum was recorded in the range of 190–260 nm at pH 7.0 and 25 °C and revealed a typical minimum at 208 nm and a shoulder at 222 nm that is characteristic mainly for predominantly α-helical proteins (Figure 4D). The deconvolution of recorded UV spectrum with the CDNN suite exhibited mainly the presence of α-helix (39.3%), followed with significant portions of β-antiparallel (6.8%) and β-parallel (7.4%) secondary structural elements, but also a rather high content of the random coil was observed (29.3%, Table 2). Global comparison of conserved secondary structure elements of selected heme peroxidases from the peroxidase-catalase superfamily is presented in Table 2. All representatives revealed a rather high content of α-helix that mainly derived from the core around the catalytic centre of heme. This is also the case for a typical hybrid B peroxidase. However, only for fungal hybrid peroxidase was there additionally a significant content of β-strands (7–9-fold higher if compared with single domain peroxidases of particular families). This observation could be probably assigned to the presence of the extra CBM-containing domain on the C-terminus of fungal hybrid B peroxidases with increased content of the β-strands [3].

**Figure 5:**
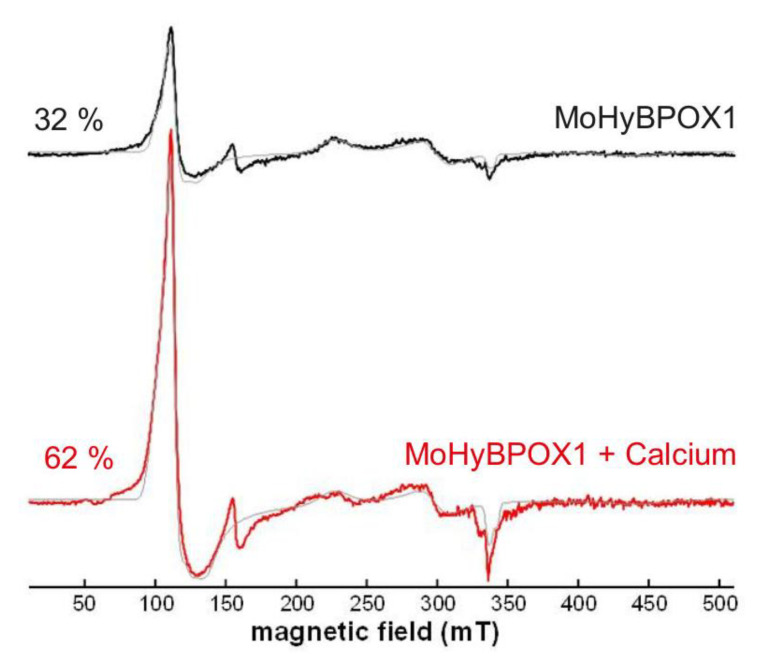
Electron paramagnetic resonance (EPR) profile of *M. oryzae* hybrid B peroxidase 1 in the ferric state showing a difference between sample of highly purified MoHyBPOX1 saturated with calcium ions (lower spectrum in red) and a comparable sample without calcium treatment (upper spectrum in black). The calculated content of high spin iron in the prosthetic heme group is presented on the left side of the diagram.

The EPR spectrum of highly purified MoHyBPOX1 in two different states is presented in Figure 5. These samples differentiated only in the presence (lower spectrum) or absence of Ca^2+^ ions (upper spectrum). Free calcium ions were proven to stabilize plant and fungal heme peroxidases [29]. Our analysis suggests for hybrid B peroxidase a mixture of high-spin (sharp peak around 100 mT) and some low-spin species (peaks around 230 mT and 280 mT) in the ferric state for both presented samples. We further calculated that for sample saturated with calcium the portion of high-spin ferric species was much higher (62% vs. 32%) thus contributing to its overall stability.

### 3.4. Unique Catalytic Properties of Hybrid B Peroxidase

The peroxidatic activity of MCAC and ion exchange purified and calcium saturated MoHyBPOX1 was recorded with 12 different one- electron donors (Table 3) at respective pH optima and at 30 °C that can be considered as the optimal temperature for the growth of this fungus. The highest specific activity was observed with tyrosine (formation of dityrosine followed fluorometrically), ABTS and 5-aminosalycilate but significant activity was also observed with L-DOPA, pyrogallol and TMB. Interestingly, not very high specific activity was detected with catechol and mainly resorcinol. A rather low level of ascorbate oxidation was observed and also a moderate specific activity of manganese (Mn^2+^ to Mn^3+^) oxidation. These two reactions are reminiscent of supposed evolutionary predecessors of hybrid peroxidases within this superfamily, namely monofunctional ascorbate peroxidases and manganese peroxidases (Figure 1). In contrast, no cytochrome *c* peroxidase activity could be determined in recorded repeat scans so such hybrid B peroxidase is definitely not an APx-C*c*P type (named so in RedoxiBase and titled previously as hybrid A peroxidases). Obtained values of specific activities can be compared with results for typical representatives of Family I (e.g., [28]), Family II (e.g., [30]) and Family III (e.g., [31] heme peroxidases). It is obvious that newly discovered phytopathogenic hybrid B peroxidase is highly active on specifically substituted phenolic compounds and aromatic amines. Among various phenolic compounds there were slight but interesting differences in the specific activities dependent on the substitutions of the aromatic ring. Due to rather high dissociation constant of the cyanide complex (Supplementary Table S2) it could be argued that most of these bulky substrates could be rapidly oxidized at the surface of the peroxidase molecule. In comparison with previous results for other heme peroxidases [30,31] it can be deduced that MoHyBPOX1 has an activity profile that fits well between Family II and Family III peroxidases (mainly due to specific activities obtained with ABTS, TMB and various phenolic compounds). Kinetic parameters for MoHyBPOX1 with those electron donors that revealed significant specific activities are summarized in Table 4. The lowest *K*_M_ value (at pH optimum) was achieved for ABTS and pyrogallol, the highest with ascorbate and manganese ions. The highest catalytic constant was recorded again with ABTS and further with L-DOPA. These results support the hypothesis that aromatic amines and phenols were probably the (best) physiological substrates of a fungal hybrid B peroxidase. However, the optimal substrates for its evolutionary predecessors, i.e., ascorbate and manganese ions were no more efficient and cytochrome c could not be oxidized at all (Table 3). The highest catalytic efficiency was achieved with ABTS that is, however, not a natural substrate. Still a rather high catalytic efficiency was recorded with L-DOPA (Table 4) and also the corresponding parameter for guaiacol was rather high and comparable with previously tested fungal lignin peroxidase [32]. Additionally, with pyrogallol a high catalytic efficiency was achieved but surprisingly not with TMB that exhibited a low catalytic constant. Here obtained kinetic results can also be compared with more recent results for Family II fungal versatile peroxidase mutants [33] or with *Pleutorus sapidus* dye decolourizing peroxidase [34] that however belongs to a different superfamily of heme peroxidases. This comparison (based mainly on parameters obtained for ABTS and guaiacol) leads to the conclusion that hybrid heme peroxidase revealed a comparably high catalytic efficiency not only with members of the same heme peroxidase superfamily but also with representatives of other heme peroxidase superfamilies that are evolutionary divergent and possess different architecture of the active centre.

### 3.5. Binding of Soluble Sugars

Sequence analysis revealed the presence of CBM motifs in all C-terminal domains of hybrid B peroxidases from Magnaporthaceae (Figure 2) so we wanted to verify the ability of these novel peroxidases to bind corresponding sugars experimentally. As was verified recently [3], the region for sugar binding was identified as CBM21 (Figure 2) that clearly belongs to starch-binding domains. Therefore, starch appeared to be the best candidate for testing on MoHyBPOX1. Results of binding experiments of soluble starch to MCAC purified and desalted MoHyBPOX1 were quantified spectrophotometrically. The peroxidase sample was incubated for a defined time with soluble starch. After its binding the protein was purified over a PD-10 column to remove unbound sugar. The colorimetric reaction monitored at 610 nm was achieved with a rapidly formed starch–I_3_ complex, and as negative controls for the possibility of non-specific protein binding of soluble sugar–BSA and cytochrome *c* (both from Sigma Aldrich) were used under the same conditions as MoHyBPOX1. Achieved results from multiple repeats of the same samples are summarized in Table 5. From the ratio of concentration of protein-bound sugar to corresponding protein concentration it was obvious that MoHyBPOX1 had 20- to 70-fold higher capacity to bind soluble starch in comparison with proteins that do not contain a carbohydrate binding motif (of CBM21 or related type).

### 3.6. Structural Peculiarities of Hybrid Peroxidases as Fusion Proteins

**Figure 6:**
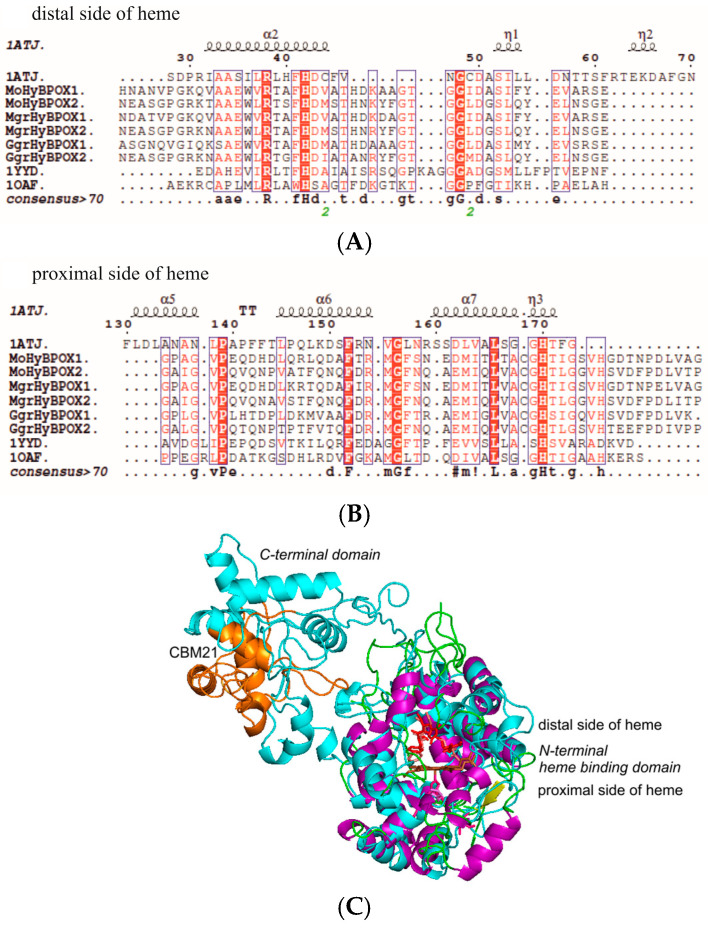
Peculiar conserved domain architecture of hybrid B heme peroxidases. Multiple structural alignment of selected HyBPOX protein sequences against the closest known experimental structure of a Family III representative (1ATJ coding for horseradish peroxidase). Regions shown: (**A**) on the distal side of prosthetic heme group and (**B**) on the proximal side of the prosthetic heme group. For comparison, also corresponding sequence regions of Family II manganese peroxidase (1YYD from *Phanerochaete chrysosporium*) and Family I ascorbate peroxidase (1OAF from *Glycine max*) are included. Performed with ESPript 3.0 [36]. Secondary structure elements from the structure of 1ATJ are indicated above the alignment. (**C**) Homology model of protein domains for MoHyBPOX1 from *Magnaporthe oryzae* performed with I-Tasser [35] in structural overlay with the template 1ATJ (horseradish peroxidase). This model was rendered with PyMOL.

The typical sequence patterns on the distal and proximal side of heme for all six here analysed fungal hybrid B peroxidases were obvious from multiple sequence alignment presented in Figure 6A,B. It is obvious that the essential distal His and Arg (around amino acid position 40 of the presented structural alignment in Figure 6A) as well as the essential proximal His (around position 170) are invariantly conserved in the same positions as for typical representatives of all three families of the peroxidase-catalase superfamily. On the distal side these two residues are involved in the heterolytic cleavage of the peroxide bond and on the proximal side conserved His coordinates the correct orientation of the prosthetic heme *b* group. Thus, we could expect a similar reaction mechanism for the novel enzymes. Further, in this figure we could compare the secondary structure elements of all six sequences of hybrid B peroxidases from the genus *Magnaporthe* with already known structural elements in typical Family II and Family III heme peroxidases. For purified MoHyBPOX1 we obtained also the far-UV CD spectrum to reveal the ratio of secondary structure elements. Obtained structural results support the evolutionary classification for hybrid B heme peroxidases between Family II and Family III (Figure 1).

In Figure 6C we see a homology model of MoHyBPOX1 performed with I-TASSER [35]. Both protein domains were modelled according to closest known homologs with experimentally known 3D structures. The structural overlay revealed a really high level of conservation for the N-terminal heme peroxidase domain in comparison with a typical Family III representative—namely horseradish peroxidase that contains only a single predominantly α-helical domain. Unique C-terminal domain of MoHyBPOX1 containing the sugar binding CBM21 region extends from the longer, mainly α-helical peroxidase domain in the region containing several loops. From this structural presentation we could deduce that the sugar binding site was located relatively far from the active site of heme peroxidase where the peroxide bond was cleaved.

From the results presented here it is obvious that hybrid B peroxidases from phytopathogenic fungi exhibited, besides significant peroxidase activity on various substrates, also the ability to efficiently bind external sugars in a separate domain. Indeed, these two quite different properties (one of them as the antioxidant qualification) constitute their real hybrid character. This duality differs them from all other known hybrid or versatile peroxidases that can only act on various electron donors. It would be therefore interesting to compare these novel oxidoreductases with other hybrid B heme peroxidases mainly from non-pathogenic fungi.

## 4. Conclusions

We presented here a typical expression pattern and basic physical and chemical properties for phytopathogenic representatives of an emerging subfamily of hybrid B peroxidases. Such novel genes coding for peroxidases of the largest superfamily are present regularly and frequently as paralogs mainly in the genomes of various fungal pathogens but also among soil as well as in endosymbiotic fungi. A functional connection of the heme peroxidase domain with carbohydrate binding motifs observed during phylogenomics analyses were systematically investigated here for the first time within a hybrid B peroxidase heterologously expressed, secreted and highly purified. The unique properties of this novel enzyme with respect to its reactivity with various peroxidase substrates might have a significant impact for the pathogenicity of the rice blast fungus (*Magnaporthe oryzae*). A concerted action of peroxide bond cleavage (peroxidase domain) and specific binding on plant cell-wall sugars (through CBM21 containing domain) could counteract with plant defence cascades based mainly on an oxidative burst and allow the phytopathogen to survive within the infected plant host tissue.

## Figures and Tables

**Table 1:** The overview on all sequenced RT-PCR products for *hyBpox* genes from *Magnaporthe oryzae* with their accession numbers in the EST database and GenBank.

Gene Identification (According to RedoxiBase)	GenBank Accessions (ID #)
*MohyBpox1*	CD037344, JZ969880; JZ969881; JZ969916; JZ969917; JZ969918; JZ969919; JZ969979; JZ970396; JZ970397; JZ970398; JZ970399; JZ970417; JZ970418
*MohyBpox2*	JZ970248; JZ970249; JZ970250; JZ970400; JZ970401; JZ970402; JZ970251

**Table 2:** Comparison of secondary structure elements deconvoluted from far-UV CD spectrum for Hybrid B peroxidase 1 from *Magnaporthe oryzae* (MoHyBPOX1) with typical representatives from all three families of the Peroxidase-Catalase superfamily.

Peroxidase Identification	α-Helix (%)	β-Strand (%)	Other (%) *
**MoHyBPOX1**—this work	39.3	14.2	46.3
**PsAPX** (PDB code: 1apx)—Family I representative	47.4	1.6	51.0
**PcMnP** (1mnp)—Family II representative	31.1	3.4	65.5
**HRP** (1atj)—Family III representative	43.5	2.0	54.5

* other elements—3_10_ helix, reverse turns and random coil.

**Table 3:** Specific peroxidase activity of secreted metal chelate affinity chromatography (MCAC)-purified MoHyBPOX1 with various electron donors at each determined pH optimum, recorded at 30 °C. All values are means ± standard deviation. For comparison, standard reduction potentials (*E*^0’^ in (V)) for used electron donors obtained from the literature are given in the second column.

Substrate	*E*^0^´ of Sub. (V)	Detection at WL (nm)	pH Optimum	Specific Activity (U/mg)
ABTS	1.08	414	6.5	217.8 ± 8.3
Guaiacol	0.43	470	6.0	8.0 ± 1.1
L-DOPA	0.47	470	6.5	103.1 ± 6.6
5-Aminosalicylate	0.54	470	6.5	188.4 ±10.3
Catechol	1.20	350	5.5	5.7 ± 0.8
Resorcinol	1.70	290	5.0	1.1 ± 0.4
Pyrogallol	0.56	430	5.5	40.2 ± 0.7
3,3,5,5-Tetramethylbenzidine	0.60	652	4.5	21.1 ± 1.5
Ascorbate	0.28	290	6.0	7.2 ± 0.1
Cytochrome *c*	0.24	550	7.0	0 *
Mn^2+^	1.51	238	8.5	7.2 ± 0.3
L-Tyrosine	0.93	fluorescence	7.5	621.6 ± 39.2 FU **

* recorded via repeat scans compared with a control sample where Cyt*c* was oxidized only non-enzymatically (i.e., scan-background); ** arbitrary fluorescence units.

**Table 4:** Michaelis–Menten parameters of purified MoHyBPOX1 for selected electron donors *.

Electron Donor	*K*_M_ (µM)	k_cat_ (s^−1^)	*k*_cat_/*K*_M_ (M^−1^ s^−1^)
ABTS	11.2 ± 1.0	106.3 ± 7.4	9.52 × 10^6^
L-DOPA	69.7 ± 2.8	24.5 ± 5.4	3.51 × 10^5^
Pyrogallol	29.3 ± 1.3	1.1 ± 0.2	3.62 × 10^4^
Guaiacol	97.3 ± 1.9	0.5 ± 0.1	5.16 × 10^3^
TMB	69.2 ± 0.7	0.2 ± 0.02	2.75 × 10^3^
Ascorbate	250.2 ± 2.3	1.2 ± 0.2	4.92 × 10^3^
Mn^2+^	762.4 ± 6.9	4.5 ± 0.3	5.96 × 10^3^

* recorded at 30 °C, at respective pH optima of tested substrates.

**Table 5:** Result of soluble starch binding on purified and desalted MoHyBPOX1 and other proteins. Presented values are means ± standard deviation.

Protein	Starch Binding (µM/µM_prot_) *
MoHyBPOX1	324.15 ± 38.04
Cytochrome *c*	4.43 ± 0.73
BSA—purified fraction	15.04 ± 1.44

* Calibration curve performed with soluble starch from Sigma Aldrich #S9765 in the same buffer as for tested protein samples.

## Supplementary Materials

The following are available online at https://www.mdpi.com/2076-3921/9/8/655/s1, Figure S1: Genomic PCR in *Pichia pastoris* and SDS-PAGE of secreted proteins. Figure S2: Mass spectrometry analysis for heterologously expressed and affinity purified MoHyBPOX1. Table S1: List of DNA primers used for the amplification of *M. oryzae hyBpox* genes. Table S2: Basic physical and chemical properties of hybrid peroxidase MoHyBPOX1.

## References

[1] Savelli B., Li Q., Webber M., Jemmat A.M., Robitaille A., Zamocky M., Mathé C., Dunand C. (2019). RedoxiBase: A database for ROS homeostasis regulated proteins. Redox Boil..

[2] Zámocký M., Hofbauer S., Schaffner I., Gasselhuber B., Nicolussi A., Soudi M., Pirker K.F., Furtmüller P.G., Obinger C. (2015). Independent evolution of four heme peroxidase superfamilies. Arch. Biochem. Biophys..

[3] Zamocky M., Janeček Š., Obinger C. (2017). Fungal Hybrid B heme peroxidases—Unique fusions of a heme peroxidase domain with a carbohydrate-binding domain. Sci. Rep..

[4] Zamocky M., Gasselhuber B., Furtmüller P.G.G., Obinger C. (2014). Turning points in the evolution of peroxidase–catalase superfamily: Molecular phylogeny of hybrid heme peroxidases. Cell. Mol. Life Sci..

[5] Reynolds K.A., Russ W.P., Socolich M., Ranganathan R. (2013). Evolution-Based Design of Proteins. Methods Enzymol..

[6] Adak S., Datta A.K. (2005). Leishmania major encodes an unusual peroxidase that is a close homologue of plant ascorbate peroxidase: A novel role of the transmembrane domain. Biochem. J..

[7] Ishikawa T., Tajima N., Nishikawa H., Gao Y., Rapolu M., Shibata H., Sawa Y., Shigeoka S. (2010). Euglena gracilis ascorbate peroxidase forms an intramolecular dimeric structure: Its unique molecular characterization. Biochem. J..

[8] Krueger T., Fisher P.L., Becker S., Pontasch S., Dove S., Hoegh-Guldberg O., Leggat W., Davy S.K. (2015). Transcriptomic characterization of the enzymatic antioxidants FeSOD, MnSOD, APX and KatG in the dinoflagellate genus Symbiodinium. BMC Evol. Boil..

[9] Zamocky M., Tafer H., Chovanová K., Lopandic’ K., Kamlárová A., Obinger C. (2016). Genome sequence of the filamentous soil fungus Chaetomium cochliodes reveals abundance of genes for heme enzymes from all peroxidase and catalase superfamilies. BMC Genom..

[10] Bellora N., Moliné M., David-Palma M., Coelho M.A., Hittinger C.T., Sampaio J.-P., Gonçalves P., Libkind D. (2016). Comparative genomics provides new insights into the diversity, physiology, and sexuality of the only industrially exploited tremellomycete: *Phaffia rhodozyma*. BMC Genomics.

[11] Smit S., Derks M.F.L., Bervoets S., Fahal A.H., Van Leeuwen W., Van Belkum A., Van De Sande W.W.J. (2016). Genome Sequence of Madurella mycetomatis mm55, Isolated from a Human Mycetoma Case in Sudan. Genome Announc..

[12] Yoshida K., Saunders D.G.O., Mitsuoka C., Natsume S., Kosugi S., Saitoh H., Inoue Y., Chuma I., Tosa Y., Cano L.M. (2016). Host specialization of the blast fungus *Magnaporthe oryzae* is associated with dynamic gain and loss of genes linked to transposable elements. BMC Genomics.

[13] Dean R., Van Kan J.A.L., Pretorius Z.A., Hammond-Kosack K.E., Di Pietro A., Spanu P.D., Rudd J.J., Dickman M., Kahmann R., Ellis J. (2012). The top 10 fungal pathogens in molecular plant pathology. Mol. Plant Pathol..

[14] Callaway E. (2016). Devastating wheat fungus appears in Asia for first time. Natural.

[15] Doehlemann G., Ökmen B., Zhu W., Sharon A. (2017). Plant Pathogenic Fungi. Microbiol. Spectr..

[16] Döhlemann G., Hemetsberger C. (2013). Apoplastic immunity and its suppression by filamentous plant pathogens. New Phytol..

[17] Solovyev V.V., Kosarev P., Seledsov I., Vorobyev D. (2006). Automatic annotation of eukaryotic genes, pseudogenes and promoters. Genome Biol..

[18] Edgar R.C. (2004). MUSCLE: Multiple sequence aligment with high accuracy and high throughput. Nucleic Acids Res..

[19] Armenteros J.J.A., Tsirigos K.D., Sønderby C.K., Petersen T.N., Winther O., Brunak S., Von Heijne G., Nielsen H. (2019). SignalP 5.0 improves signal peptide predictions using deep neural networks. Nat. Biotechnol..

[20] Pierleoni A., Martelli P.L., Fariselli P., Casadio R. (2006). BaCelLo: A balanced subcellular localization predictor. Bioinformatics.

[21] Kumar S., Stecher G., Li M., Knyaz C., Tamura K. (2018). MEGA X: Molecular Evolutionary Genetics Analysis across Computing Platforms. Mol. Boil. Evol..

[22] Ronquist F., Teslenko M., Van Der Mark P., Ayres D.L., Darling A.E., Höhna S., Larget B., Liu L., Suchard M.A., Huelsenbeck J.P. (2012). MrBayes 3.2: Efficient Bayesian Phylogenetic Inference and Model Choice Across a Large Model Space. Syst. Boil..

[23] Livak K.J., Schmittgen T.D. (2001). Analysis of relative gene expression data using real-time quantitative PCR and the 2−ΔΔC*T* method. Methods.

[24] Jacobsen N., Hensten-Pettersen A. (1970). Salivary Amylase. Caries Res..

[25] Oide S., Tanaka Y., Watanabe A., Inui M. (2019). Carbohydrate-binding property of a cell wall integrity and stress response component (WSC) domain of an alcohol oxidase from the rice blast pathogen Pyricularia oryzae. Enzym. Microb. Technol..

[26] Garcia-Arellano H., Torres E., Ayala M. (2010). Chapter 13—A Compendium of Biophysical-Chemical Properties of Peroxidases. Biocatalysis Based on Heme Peroxidases.

[27] Zamocky M., Furtmüller P.G.G., Bellei M., Battistuzzi G., Stadlmann J., Vlasits J., Obinger C. (2009). Intracellular catalase/peroxidase from the phytopathogenic rice blast fungus Magnaporthe grisea: Expression analysis and biochemical characterization of the recombinant protein. Biochem. J..

[28] Zamocky M., Droghetti E., Bellei M., Gasselhuber B., Pabst M., Furtmüller P.G., Battistuzzi G., Smulevich G., Obinger C. (2012). Eukaryotic extracellular catalase–peroxidase from Magnaporthe grisea—Biophysical/chemical characterization of the first representative from a novel phytopathogenic KatG group. Biochimie.

[29] Plieth C., Vollbehr S. (2012). Calcium promotes activity and confers heat stability on plant peroxidases. Plant Signal. Behav..

[30] Rekik H., Jaouadi N.Z., Bouacem K., Zenati B., Kourdali S., Badis A., Annane R., Bouanane-Darenfed A., Bejar S., Jaouadi B. (2019). Physical and enzymatic properties of a new manganese peroxidase from the white-rot fungus Trametes pubescens strain i8 for lignin biodegradation and textile-dyes biodecolorization. Int. J. Boil. Macromol..

[31] Krainer F.W., Pletzenauer R., Rossetti L., Herwig C., Glieder A., Spadiut O. (2014). Purification and basic biochemical characterization of 19 recombinant plant peroxidase isoenzymes produced in Pichia pastoris. Protein Expr. Purif..

[32] Koduri R.S., Tien M. (1995). Oxidation of Guaiacol by Lignin Peroxidase. J. Boil. Chem..

[33] Saez-Jimenez V., Fernández-Fueyo E., Medrano F.J., Romero A., Martínez A.T., Ruiz-Duenas F.J. (2015). Improving the pH-stability of Versatile Peroxidase by Comparative Structural Analysis with a Naturally-Stable Manganese Peroxidase. PLoS ONE.

[34] Lauber C., Schwarz T., Nguyen Q.K., Lorenz P., Lochnit G., Zorn H. (2017). Identification, heterologous expression and characterization of a dye-decolorizing peroxidase of Plerutorus sapidus. AMB Expr..

[35] Yang J., Yan R., Roy A., Xu N., Poisson J., Zhang Y. (2014). The I-TASSER Suite: Protein structure and function prediction. Nat. Methods.

[36] Robert X., Gouet P. (2014). Deciphering key features in protein structures with the new ENDscript server. Nucleic Acids Res..

