# Supplementary material for: Hybrid Heme Peroxidases from Rice Blast Fungus Magnaporthe oryzae Involved in Defence against Oxidative Stress
Source: Antioxidants (Basel). 2020 Jul 23;9(8):655. doi: 10.3390/antiox9080655 (PMC7463560; doi:10.3390/antiox9080655)
Supplement: Supplementary file 1 [file antioxidants-09-00655-s001.zip › Supplementary Figure 1.docx]

**A**


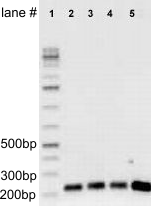


**B**


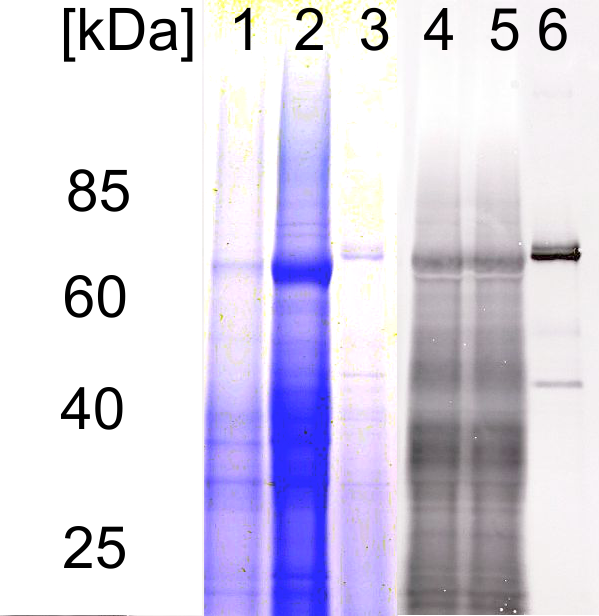


**Supplem. Figure 1.** A) Typical example for genomic PCR in *Pichia pastoris* BG11 (mutS) clones with integrated synthetic MoHyBPOX1 gene. Samples loaded on a 1% agarose gel: 1 – DNA ladder 100bp, 2 - genomic DNA from *Pichia pastoris* mutS clone MoHyB13am, 3 – clone MoHyB13fm, 4 – clone MoHyB19gm, 5 – clone MoHyB110cm; size of obtained genomic-PCR product is 220 bp. B) Samples of affinity purified MoHyBPOX1 on 4-12% SDS-PAGE with corresponding Western blot output. Lanes: 1 – after 82% ammonium sulphate precipitation Ni-MCAC pool A 2 – Ni-MCAC pool B 3 –sample additionally after DEAE Sepharose and PD-10 column 4 – same as lane 1, 5 – same as lane 2, 6 – same as lane 3. Excised bands from the sample of lane 3 in the range 60 – 70 kDa were subjected to mass spectrometry analysis presented in Figure 4.
