# Supplementary material for: Hybrid Heme Peroxidases from Rice Blast Fungus Magnaporthe oryzae Involved in Defence against Oxidative Stress
Source: Antioxidants (Basel). 2020 Jul 23;9(8):655. doi: 10.3390/antiox9080655 (PMC7463560; doi:10.3390/antiox9080655)
Supplement: Supplementary file 1 [file antioxidants-09-00655-s001.zip › Supplementary Figure 2.docx]

**A)**
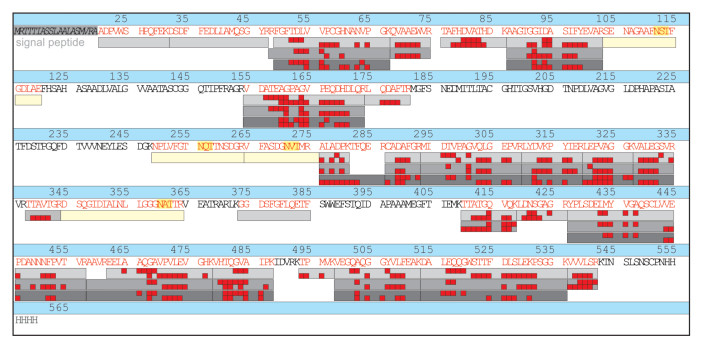


**B)**
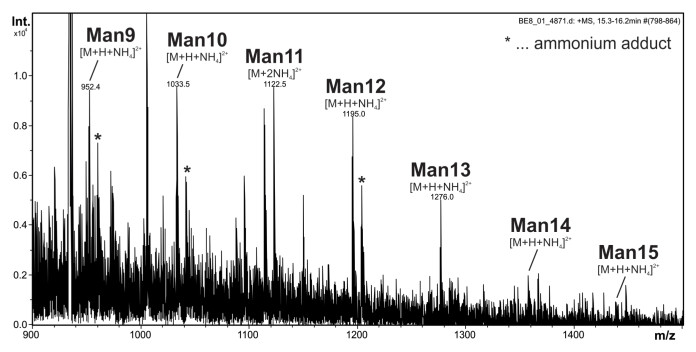


**Supplem. Figure 2.** Results of mass spectrometry analysis for heterologously expressed and affinity purified MoHyBPOX1. A) Presented is the peptide-mapping output of a major protein band excised from a SDS-PAGE gel (Suppl. Fig.1B) that was subjected to ESI-MS/MS analysis. Sequence regions indicated in red were confirmed with MS/MS fragmentation. Fragment spectra data of matched peptides is indicated as grey boxes below the sequence: fragments (b- and y-ion series) are indicated as red squares. The four N-glycosylation sites of this sequence (marked in yellow) were identified after release with PNGaseA and redigestion with Glu-C. Calculated sequence coverage is 70.74 %.

B) Obtained N-glycan spectrum of the same MoHyBPOX1 sample presented in A). The MS1 sum-spectrum shows a characteristic high mannose type glycosylation profile expected for the secretory expression in *Pichia pastoris*. N-glycans were measured on an amaZon speed ETD IonTrap (Bruker) after PNGaseA release, reduction with sodium borohydride and purification with HyperSep™ Hypercarb™ SPE Cartridges (Thermo Scientific). The molecules tend to ionize as ammonium species due to the ammonium formate buffered solvent system that was used as aqueous phase.
