# Supplementary material for: Hybrid Heme Peroxidases from Rice Blast Fungus Magnaporthe oryzae Involved in Defence against Oxidative Stress
Source: Antioxidants (Basel). 2020 Jul 23;9(8):655. doi: 10.3390/antiox9080655 (PMC7463560; doi:10.3390/antiox9080655)
Supplement: Supplementary file 1 [file antioxidants-09-00655-s001.zip › Supplementary Table 1.docx]

**Supplem.Table 1.** List of all primers used for the amplification of *M. oryzae* *hyBpox* genes.

| **Gene** | **Primer description** | **Sequence in 5´→ 3´ direction** | **Tm [°C]** |
| --- | --- | --- | --- |
| ***MohyBPOX1*** | MohyBPOX1Dfwd1 (Mor1a) | GGGTAAGAACCGCGTTCCACGAC | 62.4 |
|  | MohyBPOX1Prev1 (Mor1a) | CGATGGTGTGCCCGCATGCGGTC | 71.4 |
|  | MohyBPOX1Dfwd2 (Mor1b) | GTATCGACGCCAGCATCTTTTACGAG | 64.8 |
|  | MohyBPOX1Drev2 (Mor1b) | CTCTGGAGGTCGTGGTCTTGTTCGG | 67.9 |
|  | MohyBPOX1CtermFwd3 (Mor3) | CCAAGATCGACGTCAGGAAGAC | 62.1 |
|  | MohyBPOX1CtermRev3 (Mor3) | GTTTGGACAGCTATTTGACAGAGAATTG | 62.2 |
|  | MohyBCBMfwd4 (Mor4) | GAGACGTTTTCATGGTGGGAGTTTTC | 63.2 |
|  | MohyBCBMrev4 (Mor4) | GTCACGGGGAAGTTGTTGTTGG | 62.1 |
|  | MohyBPOX5FWD (Mor5) | GACTCGGACTTTTTCGAAGACCTC | 62.7 |
|  | MohyBPOX5REV (Mor5) | GTCGCTCTCGAGGTACTCGTTCAC | 66.1 |
| ***MohyB POX2*** | MohyBPOX2FwdA (Mor2) | CCTCCAACGAATGATAGACACTGTC | 63.0 |
|  | MohyBPOX2RevA (Mor2) | GGGATTGAAAATGTTGCCGAGTAAA | 59.7 |

**Table 3.** Basic physical and chemical properties of hybrid heme peroxidase MoHyBPOX1

| **Parameter** | **Value** |
| --- | --- |
| total length in amino acids | 548 (529 without signal peptide) |
| molecular weight of a monomer | 58149 Da (His-tagged & with heme *b*) |
| pI value (calculated, native) | 4.69 |
| signal peptide | 19 amino acids long, cleavage site between RA/AD |
| Soret maximum at pH 5.0 | 405.4 nm |
| Soret maximum at pH 6.0 | 406.6 nm |
| Soret maximum at pH 7.0 | 407.3 nm |
| Soret maximum at pH 8.0 | 408.0 nm |
| K_D_ for CN^-^ binding at pH 7.0 | 71 ± 3 µM |
