# Supplementary material for: Hybrid Heme Peroxidases from Rice Blast Fungus Magnaporthe oryzae Involved in Defence against Oxidative Stress
Source: Antioxidants (Basel). 2020 Jul 23;9(8):655. doi: 10.3390/antiox9080655 (PMC7463560; doi:10.3390/antiox9080655)
Supplement: Supplementary file 1 [file antioxidants-09-00655-s001.zip › Supplementary Table 2.docx]

**Supplem. Table 2.** Basic physical & chemical properties of hybrid peroxidase MoHyBPOX1

| **Parameter** | **Value** |
| --- | --- |
| total length in amino acids | 548 (529 without signal peptide) |
| molecular weight of a monomer | 58149 Da (His-tagged & with heme *b*) |
| pI value (calculated, native) | 4.69 |
| signal peptide | 19 amino acids long, cleavage site between RA/AD |
| Soret maximum at pH 5.0 | 405.4 nm |
| Soret maximum at pH 6.0 | 406.6 nm |
| Soret maximum at pH 7.0 | 407.3 nm |
| Soret maximum at pH 8.0 | 408.0 nm |
| K_D_ for CN^-^ binding at pH 7.0 | 71 ± 3 µM |
